# lncRNA-RNA Interactions across the Human Transcriptome

**DOI:** 10.1371/journal.pone.0150353

**Published:** 2016-03-01

**Authors:** Michał Wojciech Szcześniak, Izabela Makałowska

**Affiliations:** Department of Bioinformatics, Institute of Molecular Biology and Biotechnology, Faculty of Biology, Adam Mickiewicz University in Poznań, Poznań, Poland; CSIR Institute of Genomics and Integrative Biology, INDIA

## Abstract

Long non-coding RNAs (lncRNAs) represent a numerous class of non-protein coding transcripts longer than 200 nucleotides. There is possibility that a fraction of lncRNAs are not functional and represent mere transcriptional noise but a growing body of evidence shows they are engaged in a plethora of molecular functions and contribute considerably to the observed diversification of eukaryotic transcriptomes and proteomes. Still, however, only ca. 1% of lncRNAs have well established functions and much remains to be done towards decipherment of their biological roles. One of the least studied aspects of lncRNAs biology is their engagement in gene expression regulation through RNA-RNA interactions. By hybridizing with mate RNA molecules, lncRNAs could potentially participate in modulation of pre-mRNA splicing, RNA editing, mRNA stability control, translation activation, or abrogation of miRNA-induced repression. Here, we implemented a similarity-search based method for transcriptome-wide identification of RNA-RNA interactions, which enabled us to find 18,871,097 lncRNA-RNA base-pairings in human. Further analyses showed that the interactions could be involved in processing, stability control and functions of 57,303 transcripts. An extensive use of RNA-Seq data provided support for approximately one third of the interactions, at least in terms of the two RNA components being co-expressed. The results suggest that lncRNA-RNA interactions are broadly used to regulate and diversify the human transcriptome.

## Introduction

There are 145,331 lncRNAs known in the human transcriptome (NONCODE v4, [1]), which is over six fold more than the number of protein-coding transcripts in Ensembl 77 [2]. Up to date, however, only basic characteristics of selected lncRNAs are known. As they are highly heterogeneous in biogenesis, sequence, structure and function, the progress in deciphering their biology is relatively slow and consequently there is experimental information only for 1% of annotated lncRNAs. The data accumulated so far show that lncRNAs participate in a variety of biological processes, including transcription, splicing, translation, protein localization, cell cycle and apoptosis, imprinting, stem cell pluripotency, cellular structure integrity, and heat shock response. They have also been implicated in human diseases, e.g. it has been suggested that lncRNAs may regulate cancer progression and development [3]. lncRNAs play these roles by influencing different steps of gene expression. First of all, they can modulate the act of transcription, e.g. through promoter modifications (nucleosome repositioning, histone modifications, DNA methylation), creating a permissive chromatin environment or inhibiting the nuclear localization of specific transcription factors [4]. Such lncRNA-mediated modifications can result in activation or repression of gene expression. lncRNAs also participate in RNA processing and post-transcriptional control, which include modulation of pre-mRNA splicing, RNA editing, mRNA stability control, translation activation as well as modulating miRNA-dependent regulation [5]. It is also known that lncRNAs can act at the protein level, for instance by coming into physical interactions with alternative splicing regulators [6]. There are indications that lncRNAs can function as scaffolds to organize higher-order complexes, e.g. during histone modification [4]. Finally, lncRNAs can potentially function as signaling molecules: RNA can be transferred between cells in small vesicles known as exosomes. Because transmitted RNAs can be functional in the recipient cell, it has been suggested that lncRNAs might be involved in this mechanism and change gene expression patterns in the recipient cell [7].

**Fig 1:**
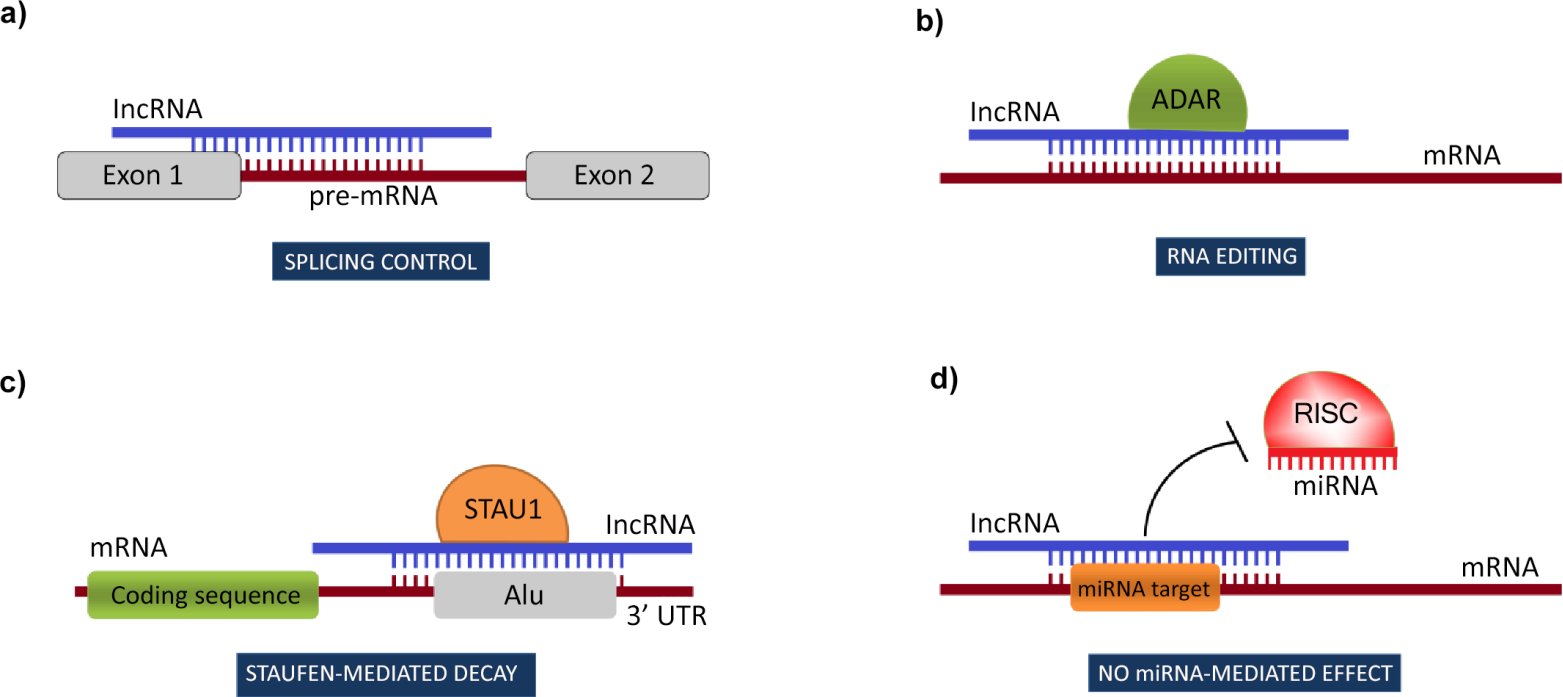
Possible roles of lncRNA-mediated interactions in transcript processing, stability control and expression regulation.

Some of the mechanisms mentioned above, like modulation of pre-mRNA splicing, RNA editing, mRNA stability control, and abrogation of miRNA-induced repression might involve interactions between long non-coding RNA and other RNA molecules. Recently published data suggest a great potential in lncRNA-mediated regulation via base-pairing with complementary RNAs but we are only starting to understand its significance. For instance, there are several lncRNAs known to play a role in splicing regulation, presumably by splice-site masking and subsequent blocking of spliceosome assembly. They require an extensive complementarity with regulated pre-mRNA molecule (Fig 1A); such complementarity occurs quite naturally between natural antisense transcripts (NATs) but interactions in *trans*, i.e. between transcripts originating from distinct loci, are possible as well. For example, NATs influence splicing patterns of mRNAs at the neuroblastoma MYC, c-ErbAalpha and ZEB2 (zincfinger E-box binding homeobox 2) loci in mammalian cells [8]. In a similar way, lncRNAs could promote hydrolytic deamination of adenosine to inosine in double-stranded RNA substrates, commonly referred to as A to I editing (Fig 1B). As inosine has the same base-pairing properties as guanosine, it pairs preferentially with cytidine instead of uridine, which alters the sequence and base-pairing properties of the edited RNA. Editing of pre-mRNAs may, for example, affect splicing–by disrupting splicing signals or creating the new ones. RNA editing of miRNA transcripts may repress their biogenesis or alter their target spectrum. On the other hand, editing of target RNA transcripts may abolish or create new miRNA-binding sites. As 85% of pre-mRNAs are predicted to undergo A to I RNA editing [9] and given that a large number of lncRNAs-RNA interactions may exist in a cell, the potential for double-stranded RNA editing in a lncRNA-dependent manner is extensive.

Finally, lncRNAs have been implicated in both positive and negative regulation of mRNA stability. For instance, Alu repeat-containing lncRNAs are involved in targeting mRNA transcripts for Staufen-mediated decay (SMD) pathway [10]. SMD is induced by Staufen 1 (STAU1) binding to double-stranded regions in mRNA 3’ untranslated regions (UTRs) (Fig 1C). Staufen-binding sites (SBSs) can be formed by intermolecular base-pairing between an Alu element within 3’ UTR of an mRNA and a partially complementary Alu element in a lncRNA. In place of Alu elements, which are unique to primates, rodents create STAU1-binding sites in presence of short interspersed elements (SINEs) of the B1, B2, B4, and ID families [11]. Importantly, in case of intermolecular RNA-RNA base-pairings, SMD targets both RNAs in the duplex, provided that they are translated. If only one RNA is translated, then it alone is targeted for SMD. As a result, lncRNA molecules, as being non-coding by definition, could affect multiple mRNAs and target them for SMD pathway. Forming lncRNA-mRNA duplexes may also result in elevated expression of mRNA molecules by masking miRNA target sites (Fig 1D). A well known example includes BACE1AS, an antisense transcript of BACE1, which competes with miR-485-5p for binding to the same region in BACE1 mRNA. This interaction has implications in Alzheimer's disease [12].

Motivated by a growing body of evidence that lncRNA-RNA interactions play crucial roles in driving transcriptome diversity, we performed a large-scale identification of human transcripts able to hybridize with lncRNA molecules. To this point, we used a similarity-search based approach with custom substitution matrix to score the alignments. Subsequent analyses showed that a number of interactions can be associated with lncRNA functions in pre-mRNA splicing, mRNA editing, Staufen-mediated decay, or modulating miRNA-dependent regulation; altogether, as many as 57,303 transcripts could be affected. We used an extensive set of RNA-Seq libraries to calculate transcript expression values, which enabled us to check whether the two components of RNA-RNA duplex are co-expressed, therefore able to interact. It occurred that in case of one third of all interactions both RNAs are co-expressed in at least one sequencing library. Taken together, our results suggest extensive roles of lncRNAs in transcript processing and gene expression regulation through base-pairing with both mRNA and pre-mRNA molecules.

## Results

### Identified lncRNA-RNA interactions and their features

The analysis revealed 15,082,791 interactions between lncRNAs and human transcripts, referred to as *lncRNA-mRNA interactions*, and 56,735,686 interactions between lncRNAs and pre-mRNAs. 3,788,306 interactions between lncRNAs and pre-mRNAs span at least one exon-intron junction and they were dubbed *lncRNA-pre-mRNA interactions*. These numbers refer to the transcript level and therefore different splice forms of a single gene may come into identical interactions with splicing isoforms of another gene. At the gene level there are 8,796,210 unique gene pairs that interact. Taking into account that 38,225 distinct Ensembl genes and 45,517 lncRNA genes were considered in the analysis, this corresponds to 0.098% of all possible gene-gene pairs. 0.21% of *lncRNA-mRNA interactions* and 0.82% of *lncRNA-pre-mRNA interactions* are in *cis* i.e. they occur between genes that occupy the same genomic loci but are transcribed from the opposite strands (Table 1). The interactions in *cis* are longer than those in *trans*, both in case of mRNAs (242 vs 186 bases, mean values) and pre-mRNAs (433 vs 237 bases, mean values). Half of the interactions are formed between lncRNAs and non-coding transcripts. Those involving protein-coding transcripts most frequently occur within 3' UTRs, constituting 39.22% and 37.76% *in lncRNA-mRNA* and *lncRNA-pre-mRNA* interactions, respectively.

**Table 1:** Comparison of selected features of mRNA and pre-mRNA interactions. In *cis* and in *trans* refer to a relative position of RNA components in a genome. CDS, 5' UTR and 3' UTR denote regions in mRNAs that base-pair with lncRNAs.

Feature	mRNA interactions (%)	pre-mRNA interactions (%)
in *cis*	0.21	0.82
in *trans*	99.79	99.18
CDS	4.10	12.01
5’ UTR	5.19	16.2
3’ UTR	39.22	6.57
CDS and UTR	2.19	9.17
non-coding	49.30	56.05

### Assessing functional consequences of lncRNA-RNA interactions

The interactions were checked against occurrences of mRNA editing sites as well as miRNA and splicing factor binding regions that could be masked by complementary lncRNA molecules. We also searched for Alu repeats within *mRNA interactions* that could bear regulatory roles.

Out of 3,788,306 *lncRNA-pre-mRNA interactions*, 2,692,097 overlap at least one alternative splice site. Altogether 21,456 alternative splice sites across human genome could be affected in this way, which involves 24,020 distinct transcripts. An example of such interactions is presented in S1 Fig. These interactions are preferentially located in non-coding transcripts (56.05%) and 5' UTRs (16.2%) as opposed to 6.57% of cases in 3' UTRs. To find out whether the interactions could be responsible for masking splicing signals, they were checked against CLIP signals for 9 splicing factors: U2AF65, PTB, FMRP, QKI, TIAL1, TIA1, HuR, TDP-43, and hnRNPC. It occurred that 27.96% of alignments that bear at least one alternative splice site overlap CLIP regions. The same is true for only 7.12% of interactions with constitutive splice sites.

Then we investigated potential regulatory roles of *lncRNA-mRNA interactions* (Fig 1B–1D). Using miRanda we identified 141,747,308 putative miRNA binding sites across 215,164 transcripts. After merging the overlapping sites and converting to genome coordinates, this corresponds to 426,727 unique regions. Next we checked these coordinates against AGO-associated CLIP data from StarBase 2.0 and we identified 271,211 common regions that constituted our final set of miRNA binding sites. We then compared them with genomic coordinates of *lncRNA-mRNA interactions*, which resulted in 21,204 regions representing putative miRNA target sites being masked by lncRNAs. Because miRNA binding sites are often shared between transcript isoforms, these genomic coordinates actually correspond to 58,698 regions across the transcriptome. Following that, we compared genomic positions of mRNA editing events from RADAR database with coordinates of *lncRNA-mRNA interactions* and we identified 82,337 regions that span at least one mRNA editing site, which corresponds to 12,853 distinct transcripts. The interactions were most often located in 3' UTRs (53.89%), followed by those located in non-coding transcripts (38.78%), 5' UTRs (4.61%) and coding regions (2.72%). Finally, using RepeatMasker we found 24,886 transcripts with Alu repeat in their 3' UTRs. In case of 7,439 of them, Alu was entirely located within lncRNA-mRNA interaction region. Such a configuration was shown to target protein-coding transcripts to Staufen-mediated decay pathway [10].

In order to estimate what fraction of identified interactions are possible in terms of the two RNA components being co-expressed, we calculated transcript expression values using 63 RNA-Seq libraries, listed in S1 Table. They include sequencing results from polyA(+) and polyA(-) libraries as well as libraries from nuclear fraction. Altogether 139,645 out of 299,154 transcripts were expressed in at least one library. In case of 36.33% of *lncRNA-mRNA interactions* and 28.84% of *lncRNA-pre-mRNA interactions* both RNA components are co-expressed in at least one library.

## Discussion

In this work we applied a similarity-based approach to identify RNA-RNA interactions across the human transcriptome. Ideally, one would apply thermodynamic functions to this task in order to calculate energies of RNA-RNA interactions and take into consideration secondary structures of both RNA components. An RNA molecule might fold in a manner that makes the complementary region inaccessible for the other component. However, the analyzed sequences are quite long (mean = 1,453 bases) and secondary structure prediction for them would be highly ineffective. The problem could be ameliorated by exploiting evolutionary conservation data for RNA structures, however lncRNAs are poorly conserved across species [13]. Moreover, data from RNA structure prediction experiments, like SHAPE, PARS and FragSeq, are by far insufficient for transcriptome-wide studies. Finally, time required when using thermodynamics-based methods makes the task infeasible. In a test that we performed on a sample of 1000 randomly selected lncRNAs-RNA pairs, it took 25.6 minutes for RNAplex [14], 23.43 minutes for RNAduplex [15], and 70.50 minutes for LncTar [16] to calculate RNA-RNA interactions, so by extrapolation it would take 1,552, 1,421, and 4.276 years, respectively, to find interactions between all considered components (215,170 lncRNAs versus 148,172 Ensembl transcripts). Our similarity-based approach reduces thermodynamics of RNA-RNA interactions to sequence similarity scoring, yet it guarantees a reasonable time for the computations; it took exactly 41 minutes and 29 seconds on a single CPU to complete the task in a transcriptome scale. Keeping this in mind, we recently used this approach to identify lncRNA-RNA transcriptome-wide interactions in ten plant species [17].

To test the accuracy of our pipeline, we followed a recently proposed method, with modifications [16]. First, we generated a set of 5000 random NONCODE v4 lncRNAs and the same number of Ensembl transcripts and we associated them into 5000 lncRNA-RNA pairs. We then tested the sequences with our pipeline and as a result 9 out of the 5000 random lncRNA-RNA pairs have been found as valid associations. This gives the specificity of 99.82%, supposing the identified interactions are indeed not existent in a cell. As a positive data set we used ten manually curated lncRNA-RNA interactions [16] and we were able to recover 80% of them. The two missing interactions were mediated by a single lncRNA, called BC200 or NONHSAT070572 at NONCODE v4. This gives much higher specificity than in case of LncTar (99.82% vs 95%) at the same sensitivity. However, it is important to note that the positive dataset, comprising of only 10 cases, is too small to reliably assess the sensitivity of both approaches, yet we are limited to these positive cases because of data availability; even databases that are supposed to collect RNA-RNA interactions, likeRAID [18], most often store associations that are based on similar functionalities of the two RNA molecules, their co-localization or coexpression, rather than experimentally supported RNA-RNA base-pairings.

In order to support the predictions with transcriptomic data, we calculated transcript expressions values across 63 human RNA-Seq libraries, spanning a number of human tissues and cell lines. Products of RNA polymerase II possess a polyA tail at their 3' end, a feature being exploited in RNA-Seq technology to deplete ribosomal RNA fraction in the sequencing library. However, a number of non-coding transcripts are products of RNA polymerase III and lack polyA tail. Therefore, we took advantage of 27 RNA-Seq libraries without polyA selection, i.e. where RiboMinus depletion of rRNA was applied. Altogether, out of 299,154 distinct human transcripts (lncRNAs from NONCODE and Ensembl transcripts), 139,645 (46.68%) were expressed in at least one library. 24.1% of transcripts were expressed in libraries without polyA selection but only 1.7% of all sequences were expressed uniquely in these libraries. Next, we found that in case of 5,480,029 (36.33%) *lncRNA-mRNA interactions* and 1,092,516 (28.84%) *lncRNA-pre-mRNA interactions* both RNA components are co-expressed in at least one library. These numbers suggest that a large proportion of found interactions may indeed exist in a cell. It might be, however, that the values are underrated, as we applied quite stringent criteria for expression calculation, taking into consideration only those sequencing reads that map uniquely to a given transcript, especially that analyzed genes have on average 6.62 isoforms and they overlap extensively. For the same reason the actual number of transcripts that get transcribed might be significantly higher. Then we calculated the expression values in a modified manner, where a read was required to map uniquely to a gene (instead of a transcript). With this approach we were able to detect expression of 170,798 (57.09%) transcripts. However, this procedure, alike other popular approaches, e.g. RSEM [19], does not guarantee that a particular transcript is indeed expressed. Last but not least, one should consider relative expression values of RNA molecules that interact; ideally, they would be expressed at similar levels or the regulatory molecule (here, lncRNA) would be in excess. However, due to abovementioned issues with estimation of transcript expression values, these calculations cannot be performed in a confident way, although we checked that over 96% of co-expressed molecules meet the following criteria i) less than 10-fold difference in expression values or ii) lncRNA is expressed in excess.

The interactions are predominantly found in *trans* and only 0.21% and 0.82% of them are in *cis*, in *lncRNA-mRNA* and *lncRNA-pre-mRNA* interactions respectively. A higher proportion of *cis* interactions in the latter group can be explained by the fact that transcripts being close each other in a genome have higher chance to interact and, additionally, they base-pair without mismatches, thus providing higher hybridization energies. Both features might be critical for efficiency of base-pairing and could have functional consequences in splicing regulation, owing that this is a fast process, occurring co-transcriptionally. Regarding protein-coding transcripts, the *lncRNA-mRNA interactions* are predominantly located in 3' UTRs (39.22%) versus 5.19% in 5' UTRs, 4.10% in coding sequences and 2.19% spanning both CDS and UTR; the remaining interactions are found in non-coding transcripts (49.30%). This observation can be partially explained in terms of region lengths. For example, the cumulative length of all human 5' UTRs and 3' UTRs is 20,989,925 and 73,183,804 nucleotides respectively, thus there are higher chances a lncRNA will interact with a 3' UTR. Moreover, 3' UTRs bear higher proportion of repetitive elements (11.88%) than 5' UTRs (8.88%) and the whole transcriptome (8.43%), which adds up to the preference of interactions to occur in 3' UTRs, especially that lncRNAs possess even higher proportion of repeats (25.83%). Notably, the observed preference to base-pair with 3’UTRs is exactly what one would expect from the biological point of view, as 3' UTRs play critical roles in gene expression regulation. Still, however, in case of lncRNA-mRNA interactions spanning miRNA binding sites, there is preference towards coding sequences (37.59%), compared to only 29.78% of interactions located in 3’UTRs. We postulate this could be linked to lncRNA functions in preventing RISC complex from binding to the coding regions, leading to increased target specificity of microRNAs. In *lncRNA-pre-mRNA interactions*, alignments within 3’ UTRs (6.57%) are outnumbered by those within 5’ UTRs (16.2%). This cannot be simply explained by the requirement that alignments span splice sites, because generally almost equal numbers of splice sites can be found within 5’ and 3’ UTRs in transcripts of interest, 34,949 and 33,141 respectively. Keeping in mind that protein-coding transcripts have almost eightfold more introns in 5' UTRs than in 3' UTRs, while disfunctional transcripts, like those targeted for nonsense-mediated decay (NMD) pathway, tend to have an elevated number of introns downstream coding region, we hypothesize that the preferential occurrence of interactions within 5' UTRs may indicate involvement of lncRNAs in RNA stability control mechanisms in a cell.

Our analysis indicated altogether 57,303 transcripts that could be regulated by means of lncRNA-RNA interactions. This would be achieved in the following ways: i) masking splice sites and other splicing signals, ii) competing with miRNAs for binding sites on transcripts, iii) promoting mRNA editing events, and iv) hybridizing with 3' UTRs of Alu-containing transcripts and targeting such mRNA molecules for degradation through Staufen-mediated decay pathway. S1 File lists all human transcripts predicted to be subject to this kind of regulation. It needs to be stressed that consequences of lncRNA-RNA base-pairing might go far beyond these mechanisms as we only considered several scenarios here. Moreover, the found interactions need more insight and laboratory tests to provide experimental support for them and learn about their biological significance. Still, however, our results indicate a great potential in lncRNA-mediated regulation and they constitute an important step towards deciphering functions of long non-coding RNAs.

## Methods

### Data download

Genome and transcriptome sequences as well as corresponding annotation data were retrieved from Ensembl 75 [2] using BioMart and the download page. lncRNA sequences and their genomic coordinates came from NONCODEv4 [1]. AGO and transcription factor binding sites from CLIP experiments were downloaded in BED format from StarBase 2.0 [20]. mRNA editing sites were obtained from RADAR database [21] in BED format. Fastq files from 63 RNA-Seq libraries were downloaded from European Nucleotide Archive [22], listed in S1 Table. Finally, mature miRNA sequences came from miRBase Release 21 [23].

### Identification of lncRNA-RNA interactions

We used *lastal* from LAST package [24] to identify potential lncRNA-RNA interactions. To this point we created a custom substitution matrix that enabled us to search for G:U (wobble) pairs (S2 File). The reason why we used *lastal* is that other popular similarity-search tools, like BLAST, do not allow user-supplied scoring matrices, although BLAST has already been used in a similar task [25]. In the substitution matrix, G:C, A:T and G:T matches were scored 4, 2 and 1, respectively. These proportions have been widely used in the field of RNA-RNA interactions, e.g. in miRNA target search algorithms [26]. Additionally, a mismatch was scored -6, gap opening -20 and gap extension -8. *Lastex*, from the same package, enabled us to estimate a threshold value for alignment scores. We set this threshold to 107, which corresponds to less than one alignment expected to occur by chance. In the interaction search procedure, lncRNAs from NONCODE constituted a database, while transcript sequences were used as a query. When looking for lncRNAs base-pairing with pre-mRNAs, the query transcripts contained intron sequences; any intronic sequences distant by more than 250 bases from 3' or 5' splice sites were masked with *N* characters using Python scripts and based on exon coordinates from Ensembl. Here, the threshold value for alignment score was set to 108, as estimated with *lastex*. Having the *lastal* results in MAF format, we applied a set of in-house Python scripts to process the data and prepare them for subsequent steps: (i) the coordinates of intermolecular alignments were converted to genomic positions, (ii) the coordinates were merged to unique positions in the genome, (iii) the MAF files were converted to BED format.

### Retrieving interactions that could be involved in regulatory processes (Fig 2)

#### Splicing regulation through masking splicing signals

Interactions between lncRNAs and pre-mRNAs were filtered to keep only those that spanned exon-intron borders. These alignments, after converting to BED format with genomic positions, were superimposed with StarBase 2.0 coordinates for human splicing factors. It was required that both features occupied the same DNA strand and putative splicing factor binding site was located entirely within the interaction region. Alternative splice sites were identified based on exon coordinates from Ensembl using Python scripts.

**Fig 2:**
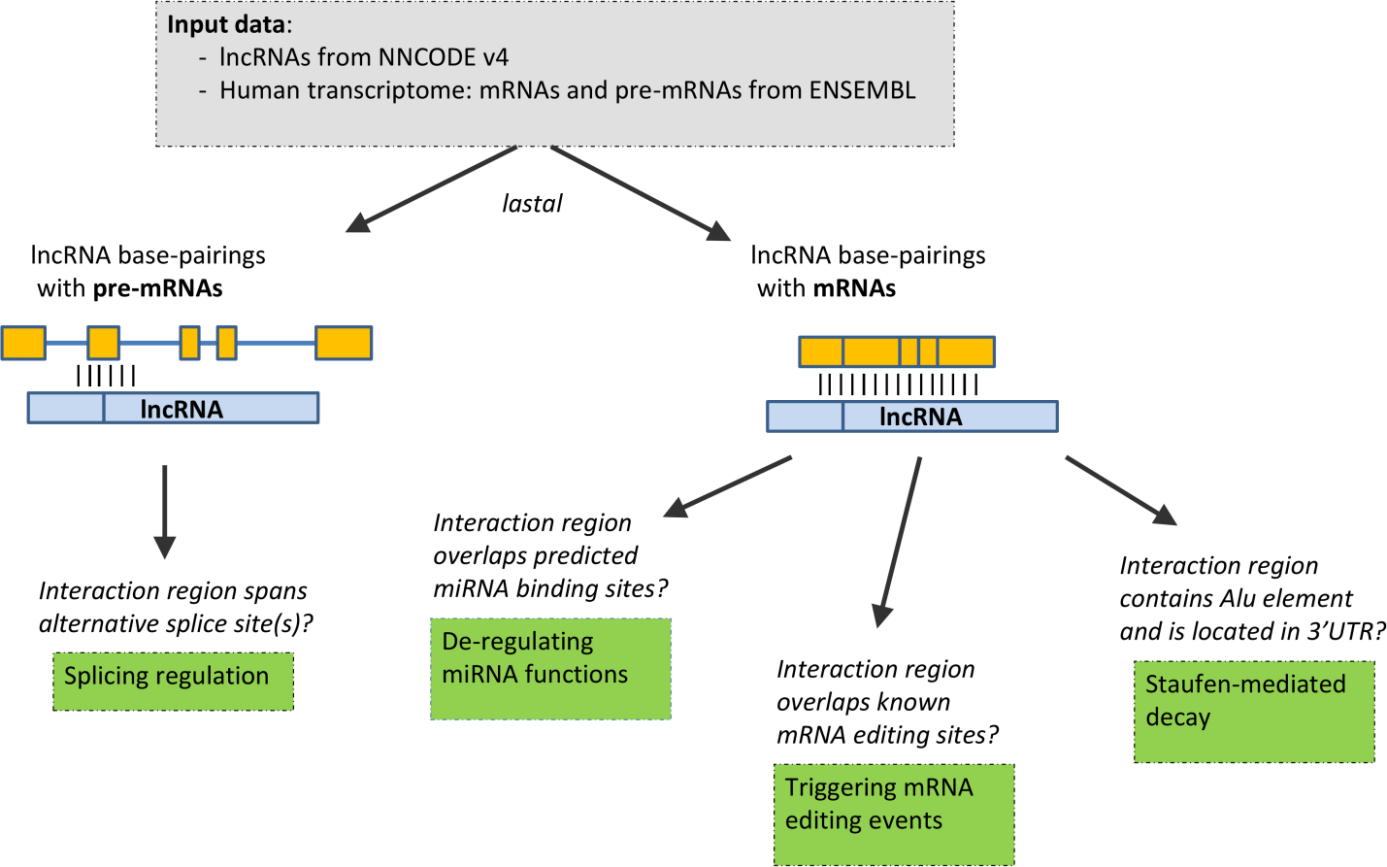
A summary of our approach aiming at identification of regulatory lncRNA-RNA interactions.

#### Abrogation of miRNA-dependent regulation

StarBase 2.0 coordinates of putative miRNA binding sites were put into a single file and converted to non-overlapping coordinates with BEDTools *merge* [27] tool to remove data redundancy. Then, to provide further support for these target sites and annotate them, miRNA target prediction with miRanda [28] was performed using default settings. The two datasets i.e. miRNA binding sites from CLIP-Seq experiments (Starbase 2.0) and those from miRanda predictions, were superimposed and the common part was checked against lncRNA-mRNA alignments. It was required that at least half of the miRNA target site was located within the alignment. Obtained in this way miRNA targets were considered as potentially masked by hybridizing lncRNA molecules.

#### lncRNAs involved in Staufen-mediated decay (SMD) pathway

Coordinates of Alu elements in human transcripts were identified with RepeatMasker (http://www.repeatmasker.org). They were required to be entirely located within the lncRNA-mRNA alignment region and additionally in a 3' UTR of protein-coding transcripts.

#### Triggering mRNA editing events

lncRNA-mRNA alignments were converted to BED format with genomic coordinates and checked against mRNA editing sites from RADAR database. Any adenine to inosine editing event located within the interaction region was considered as potentially triggered by lncRNAs.

### Further data processing and analysis

To calculate transcript expression values, RNA-Seq reads were mapped with Bowtie [29] to the non-redundant set of sequences from Ensembl 75 and NONCODE v4 with one mismatch allowed. Reads mapping to more than ten positions were discarded. Then, using in-house Python scripts, reads that mapped to more than one gene were removed and RPKM values for transcripts were calculated using reads that uniquely mapped to a transcript.

Repetitive sequences were identified with RepeatMasker 4.0.0. BEDTools suite [27] was applied for operations on BED files. In particular, BEDTools *intersect* was used to identify overlapping regions between two datasets of interest, while BEDTools *merge* generated non-overlapping sets of feature coordinates.

## Supporting Information

S1 Fig A selected example of lncRNA-RNA interaction. a) lncRNA NONHSAT001705 base-pairs with pre-mRNA of ENST00000413854 in a way that the alignment spans an alternatively spliced exon as well as parts of surrounding introns. b) NONHSAT001705 gets expressed predominantly in white blood cells and SK-N-SH cell line, which was originally isolated from a bone marrow. On the other hand, ENST00000413854, a transcript of RHCE gene coding for Rh blood group antigens is located on chromosome 1 close to NONHSAT001705, suggesting the interaction could have functional consequences.
(PDF)

S1 File A list of human transcripts whose processing, fate and expression levels might be under control of lncRNAs. The file contains a list of all human transcripts putatively affected by lncRNAs through one of the following mechanisms: i) masking splice sites and other splicing signals, ii) competing with miRNAs for binding sites on transcripts, iii) promoting mRNA editing events, and iv) hybridizing with 3' UTRs of Alu-containing transcripts and targeting mRNA molecules for degradation through Staufen-mediated decay pathway.
(TXT)

S2 File A substitution matrix used to score alignments with *lastal*. The matrix is constructed in a way that enables identification of G:T (G:U) pairs, which is distinct from default *lastal* behavior.
(TXT)

S1 Table A summary of 63 RNA-Seq libraries used to estimate transcript expression values. (XLSX)
