# Supplementary material for: lncRNA-RNA Interactions across the Human Transcriptome
Source: PLoS One. 2016 Mar 1;11(3):e0150353. doi: 10.1371/journal.pone.0150353 (PMC4773119; doi:10.1371/journal.pone.0150353)

a)

```
ccagctcat intron

GTGTCACCT exon

NONHSAT001705   GGTCGAGTAAGGGATTTACGACGTGTTAGTCCCATTGACACGGGACTCGGATTCTCCGTCATCACTCGACCGGGTAGTACAGGTGACTACTTCCTGTGCATCGGGGTTGTGTCCCCTCTTCACCAAAGTCCTAGTCGTTTCGTCCCTCCT
                ||||||||||||||||||||||||||||||||||||||||||||||||||||||||||||||||||||||||||| |:|||||||||||||||||||||||||||||||||||||||||:||||||||||||||||||||||||||||||
ENST00000413854 ccagctcattccctaaatgctgcacaatcagggtaactgtgccctgagcctaagaggcagtagtgagctggcccaccgtgtccactgatgaaggacacgtagccccaacacaggggagaggtggtttcaggatcagcaaagcagggagga

NONHSAT001705   ACAATGTCCCAACGGAACAAGGGTCGCACGACCAGTGAACGTCGTTCTACCACAAGAGAGAGATGGAACGAAGGAAATGGGTGTGCGATAAAGAAACGTCTGAATACACGTGTCACGCCACAACCGTCCTCCGCACCGACACCCATGGAG
                ||||||||||||||||||||||||||||||||||||||||||||||||||||||||||||||||||||||||||||||||||||||||||||||||||||||||||||||||||||||||||||||||||||||||||||||||||||
ENST00000413854 tgttacagggttgccttgttcccagcgtgctggtcacttgcagcaagatggtgttctctctctaccttgcttcctttacccacacgctatttctttgcagACTTATGTGCACAGTGCGGTGTTGGCAGGAGGCGTGGCTGTGGGTACCTC

NONHSAT001705   CACAGTGGACTAGGGAAGAGGCACCGAACGGTACCACGACCCAGAACACCGACCCGACTAGAGGCAGCCCCCTCGGTTCATGGACGGCCATTCTTTGATCTGTTGATTGGAGGAGACGAAACCGACTTCCGGTCGTCCTGCGACCCTGGA
                |||||||||||||||||||||||||||||||||||||||||||||||||||||||||||||||| |||||||||||||||:|||||||||||||||||||||||||||:  :||||||||||||||||||||||||||||||||||||||
ENST00000413854 GTGTCACCTGATCCCTTCTCCGTGGCTTGCCATGGTGCTGGGTCTTGTGGCTGGGCTGATCTCCATCGGGGGAGCCAAGTGCCTGCCGgtaagaaactagacaactaatgctctctgctttggctgaaggccagcaggacgctgggacct

NONHSAT001705   CTACCCGGTGACACGTCACGTGTCGACGTAATCCGTCCACAGCCGCGTAAGAGAATAACCGAAGTTGCGGATCACTCCCTAGGTAGGACCGAGCCACCGCGTAAACAATTCTACGAGCCCTCGTCCACCGTCTTGGGTAAACTCGAACGA
                |||||||||||||||||||||||||||||||||||||||||:||:|||||||||||||||||||||||||||| ||||||||||||||||||||||||| |||||||||||||||| |||||||||||||||||||||||||||||||||
ENST00000413854 gatgggccactgtgcagtgcacagctgcattaggcaggtgttggtgcattctcttattggcttcaacgcctagcgagggatccatcctggctcggtggcacatttgttaagatgctggggagcaggtggcagaacccatttgagcttgct

NONHSAT001705   ACCCGTAACCCCTCTTAAACAATAGTCCGATGACCCCA
                |||||| |||||||||||||||| |||||||| |||||
ENST00000413854 tgggcactggggagaatttgttaccaggctacaggggt
```

b)

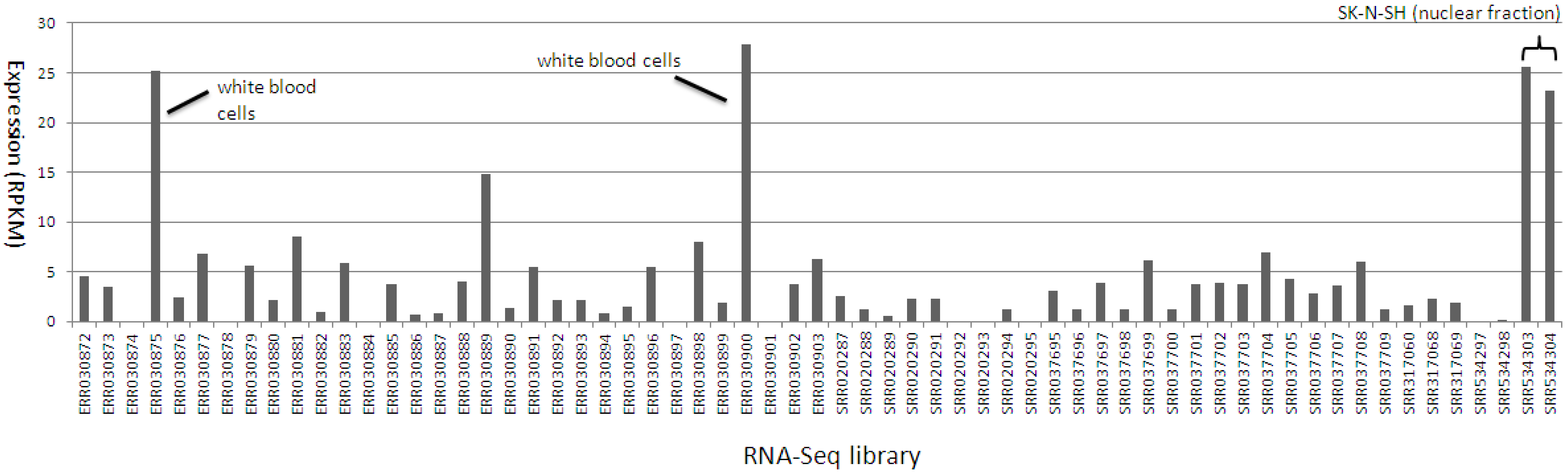

Supplement: S1 Fig — a) lncRNA NONHSAT001705 base-pairs with pre-mRNA of ENST00000413854 in a way that the alignment spans an alternatively spliced exon as well as parts of surrounding introns. b) NONHSAT001705 gets expressed predominantly in white blood cells and SK-N-SH cell line, which was originally isolated from a bone marrow. On the other hand, ENST00000413854, a transcript of RHCE gene coding for Rh blood group antigens is located on chromosome 1 close to NONHSAT001705, suggesting the interaction could have functional consequences. (PDF) [file pone.0150353.s001.pdf]
